# Plasmodium falciparum malaria and Parvovirus B19; a case of acute co-infection

**DOI:** 10.1186/1471-2334-10-87

**Published:** 2010-04-01

**Authors:** F Ingrassia, A Gadaleta, P Maggi, G Pastore

**Affiliations:** 1 Institute of Infectious Diseases, University of Bari, Italy, Ospedale Policlinico Piazza Giulio Cesare, 30, 70124 - Bari, Italy

## Abstract

**Background:**

Co-infection with Plasmodium falciparum malaria and Parvovirus B19 in adults is an extremely rare occurrence and, apparently, only one case has been previously reported. Herein we describe a case of acute co-infection with severe anemia and renal failure.

**Case presentation:**

The patient was a 34-year-old African man presenting myalgia, fatigue, headache, anemia and hepatosplenomegaly. A thin peripheral smear showed Plasmodium falciparum trophozoites and the patient was treated with oral mefloquine. After an initial amelioration, fever, fatigue and myalgia reappeared, the anemia worsened and there was evidence of acute renal failure. No malarial parasites were found with a blood smear. A bone marrow aspiration showed marked erythroid hypoplasia. Parvovirus B19-specific IgM and IgG and viremia were positive. The patient was treated with steroids and blood cell transfusions. After ten days, anemia and renal failure progressively decreased. When last seen, the patient was asymptomatic and the blood values were within the normal range.

**Conclusions:**

The diagnosis of Parvovirus B19 acute infection should be considered in any case of persistent severe anemia and/or renal failure, even in clinical conditions that are well-known causes of anemia and renal failure, such as malaria.

## Background

Parvovirus B19 is the etiologic agent of erythema infectiosum (fifth disease), although it may also be associated with transient aplastic anemia. Malaria due to *Plasmodium falciparum *is a protozoan disease characterized by fever with chills and hepatosplenomegaly. Possible complications are cerebral malaria, anemia, haemoglobinuria, acute renal failure, and spleen rupture.

Co-infection of *Plasmodium falciparum *malaria with Parvovirus B19 in adults is a very rare occurrence and, to the best of our knowledge, only one case has been reported in the literature [1].

Therefore, both these pathogens are potent causes of anemia and a co-infection could determine life-threatening anemia. Herein we describe a case of acute co-infection in an adult patient with severe anemia and renal failure.

## Case presentation

The patient was a 34-year-old African man immigrated from Ghana and living in Italy for twenty years. He returned to his native country in March 2007 for three weeks, and one month after his return, he was hospitalized with high fever (up to 39°C), intense myalgia, fatigue, and headache. His was 190 cm high and weight 100 kg. A physical examination revealed only hepatosplenomegaly with a 2 cm liver enlargement and a 4 cm spleen enlargement. Blood pressure was 110/60 mmHg.

A complete blood count showed the following pathological values: hemoglobin 11.3 g/dl, total white blood cells 5.100/mm3 (61.8% neutrophils, 29.8% lymphocytes and 7.3% monocytes). The reticulocyte cell count was 3,65% and platelet count was 39.000 mm3. A mild increase in transaminase levels was observed (ALT 84 IU/l, AST 102 IU/l). Creatininemia and blood urea were within normal limits (respectively 0.9 mg/dl and 35 mg/dL). Antibodies to *Salmonella typhi*, Brucella spp., Leishmania spp., Rickettsia, Cytomegalovirus and Epstein-Barr virus were not detected; urine and stool cultures were negative.

A thin peripheral smear showed *Plasmodium falciparum *trophozoites (parasitemia 5%). The patient was treated with oral mefloquine at standard doses. After four days fever subsided and parasitemia became undetectable. However, two days later, fever, fatigue and myalgia reappeared.

Haemoglobin level decreased to 7.1 g/dL, reticulocytes were reduced to 0.4%, and there was evidence of acute renal failure with creatininemia and azotemia levels of 5 mg/dl and 143 mg/dl, respectively. Chest X rays revealed pleural effusion. No malarial parasites were found on blood smear.

In light of this clinical situation, a bone marrow aspiration was performed which showed marked erythroid hypoplasia. Parvovirus B19 specific IgM and IgG (*Bioelisa Parvovirus B19 *Instrumentation Laboratory, Milan, Italy) together with Parvovirus B19 viremia (*COBASAmpliPrep*, Roche Diagnostics GmbH, Mannheim, Germany) were positive. Due to the persistence of anaemia and the evidence of bone marrow hypoplasia, the patient was treated with steroids for the thrombopenia, and blood cell transfusions (three units). After ten days, the hemoglobin level was 11.7 g/dL, and signs of renal failure progressively decreased (creatininemia 1.8 mg/dl and azotemia 60 mg/dl). A search for antibodies to Parvovirus B19 was repeated, and the diagnosis of acute Parvovirus B19 infection was confirmed (IgM became negative, while IgG increased to higher titers. After a further 20 days without steroid therapy, the patient was asymptomatic and the blood values were within the normal range.

The patient gave informed consent for the processing of his data.

Parvovirus B19 is the etiologic agent of erythema infectiosum (fifth disease), a common benign and usually self-limited childhood exanthema, although it may be associated with transient aplastic anaemia, especially in patients with chronic haemolytic anemia and/or in immunocompromised hosts. Indeed, erythroid precursor cells represent the common target of Parvovirus B19 [2-7].

When acute Parvovirus B19 infection occurs together with other infections or clinical conditions associated to haemolytic anaemia and/or dyserythropoiesis such as malaria, as in the present case, the respective pathogenetical mechanisms leading to anemia could be synergistic, thus further reducing the haemoglobin levels.

Anaemia in malaria can be associated with more pathogenetic mechanisms, such as haemolysis and dyserythropoiesis. Erythroid precursor cells represent the common target for Parvovirus B19 and Plasmodium falciparum and the co-infection may cause severe anemia up to acute aplastic crises [2-7]. In the literature, only one case is reported of coinfection with malaria and Parvovirus B19 in a47 year-old Italian tourist returning from Mali [1]. Similarly to our case, also fever and severe anemia persisted after treatment for malaria was discontinued, although renal failure was not evidenced.

Indeed, recent reports indicate a possible role for Parvovirus B19 in 'idiopathic' collapsing focal segmental glomerulosclerosis, leading to acute renal failure [8-10]. The pathogenetical mechanisms of renal involvement during Parvovirus B19 infection are unclear and can include immune complexes deposition, direct cytopathic effects, and autoantibody production [10].

If Parvovirus can determine, as documented above, an anemia due to erythroid hypoplasia, anemia in malaria can largely involve haemolysis. Moreover, also malaria is a well-known cause of acute renal failure. In our case, acute renal failure appeared several days after malaria resolution, together with detection of IgM to Parvovirus B19; in addition, the resolution of the complication occurred at the moment of IgM clearance. Thus, we can hypothesize that, even if acute renal failure could be determined by the co-infection, Parvovirus B19 probably played a major role in its onset.

## Conclusions

In conclusion, in the light of the present experience, the diagnosis of Parvovirus B19 acute infection should be considered in any case of persistent severe anemia and/or renal failure, even in clinical conditions that are well-known causes of anaemia and renal failure, such as malaria.

## Competing interests

The authors declare that they have no competing interests.

## Authors' contributions

F.I., A.G. followed the patient from the clinical point of view performed the follow-up and drafted the manuscript; G.P. and P.M. supervised and coordinated the paper. All authors read and approved the final manuscript.

## Pre-publication history

The pre-publication history for this paper can be accessed here:

http://www.biomedcentral.com/1471-2334/10/87/prepub
